# Transient Overexpression of the Pepper *WRKY2* Gene in *Nicotiana benthamiana* Markedly Delays the Systemic Necrosis Caused by Tobacco Mosaic Virus

**DOI:** 10.3390/life15040669

**Published:** 2025-04-17

**Authors:** Csilla Juhász, Ágnes Szatmári, Zoltán Bozsó, Balazs Barna, Gábor Gullner

**Affiliations:** Plant Protection Institute, HUN-REN Centre for Agricultural Research, Fehérvári Str. 132-144, 1116 Budapest, Hungary; csilla.juhasz11@gmail.com (C.J.); agiszatmari@gmail.com (Á.S.); bozso.zoltan@atk.hun-ren.hu (Z.B.);

**Keywords:** pepper, WRKY, transcription factors, virus resistance, tobacco mosaic virus

## Abstract

The role of WRKY transcription factor proteins in plant defense reactions against fungal and bacterial pathogens is well studied, but less information is available about plant–virus interactions. We observed the rapid and strong activation of the transcription factor gene, *CaWRKY2*, in pepper leaves following inoculation with Obuda pepper virus (ObPV). In contrast, *CaWRKY2* was only weakly induced by pepper mild mottle virus (PMMoV) inoculation. To carry out a functional analysis of *CaWRKY2*, the gene was transiently overexpressed in *Nicotiana benthamiana* leaves by agroinfiltration. Four days later, *CaWRKY2*-overexpressing and empty vector control leaves were inoculated with tobacco mosaic virus (TMV). Transiently overexpressing *CaWRKY2* did not affect the replication rate of TMV in the inoculated leaves. However, TMV inoculation up-regulated the expression of a pathogenesis-related gene (*NbPR-1b*) and a lipoxygenase (*NbLOX1*) gene significantly more strongly in *N. benthamiana* leaves overexpressing *CaWRKY2* than in empty vector control leaves. Intriguingly, *CaWRKY2* overexpression delayed (by 3 days) the development of systemic necrosis and plant death caused by TMV in *N. benthamiana*. These results suggest that CaWRKY2 is able to hinder the spread of TMV from inoculated leaves towards vascular tissues and systemic leaves in *N. benthamiana*.

## 1. Introduction

In virus-infected plants, numerous biochemical defense reactions are activated. If these reactions are robust and rapid, virus infection is unsuccessful (incompatible interaction or resistance). In resistant plants, the invading virus is rapidly perceived by cell surface receptors or by resistance proteins (R-proteins) [1]. Upon recognition, signals are transmitted to the nucleus mainly by kinase cascades, leading to the extensive reprogramming of gene expression patterns and ultimately to the production of antimicrobial proteins and metabolites [2,3]. On the other hand, during compatible plant–virus interactions (in susceptible plants), only late and weak host defense reactions occur, which allow the rapid multiplication and systemic spreading of the virus [4,5].

The reprogramming of host plant transcriptome is regulated by a complex, multilayered regulatory network in which various transcription factors play critical roles [6]. WRKY transcription factors are promising research targets due to their involvement in various plant–pathogen interactions [7,8,9]. WRKYs are encoded by large multigene families in plants. Their protein sequences contain one or two characteristic Trp-Arg-Lys-Tyr (WRKY) amino acid motif(s) and zinc finger motif(s). WRKYs are classified into three groups, and the large one, group II, is further divided into five subgroups according to the number of WRKY motifs and the type of zinc finger motifs they have [7,10]. WRKYs specifically recognize and bind to the W-box motif [canonical sequence: (C/T)TGAC(C/T)] or to its variants within the promoters of target genes [7,11]. The binding of WRKY proteins to the promoters of their numerous target genes can positively or negatively regulate their transcription. Intriguingly, W-box DNA elements can also constitutively be occupied by WRKY proteins in untreated plants [12].

In pepper (*Capsicum annuum* L.), the role of WRKYs has been extensively studied. The genome-wide identification of pepper *WRKY* genes has been carried out by several research groups, which caused some trouble in the nomenclature of *WRKYs*. In different reports, 71, 61, 62, and 72 *WRKY* genes were identified in the pepper genome [13,14,15,16]. Considerable attention has been focused on the role of WRKYs during different pepper–tobamovirus interactions due to the economic importance of these viruses. The early up-regulation of *WRKY-a*, *WRKY-b*, and *WRKY-d* genes was observed during the incompatible interaction between pepper and the P_0_ strain of tobacco mosaic virus (TMV) [17,18,19]. In addition, the marked induction of *CaWRKY1* and *CaWRKY2* genes was observed during the incompatible interaction between *Capsicum chinense* harboring the *L*^3^ resistance gene and the P_1,2_ pathotype of pepper mild mottle virus (PMMoV) [20,21]. In compatible interactions between pepper and tobamoviruses, the induction of *WRKY* genes was usually negligible [17,19]. Functional studies that elucidate the exact role of pepper WRKYs are scarce. Gene silencing experiments proved that *WRKY-a*, *WRKY-b*, and *WRKY-d* are positive regulators of antiviral defense [8,18,19]. On the other hand, the overexpression of *CaWRKY1* in transgenic *Nicotiana tabacum* cv. Xanthi-nc (genotype NN) plants resulted in diminished resistance against TMV [21].

Previously, we found that the inoculation of pepper leaves with Obuda pepper virus (ObPV) led to the appearance of hypersensitive necrotic lesions, while a PMMoV strain caused only very mild chlorotic symptoms [22,23]. ObPV strongly induced the expression of fatty acid desaturase, lipoxygenase, and divinyl ether synthase genes [24,25,26]. In addition, ObPV led to a strong accumulation of defense hormones [27] and elevated glucose, fructose, and glucose-6-phosphate levels [28]. Recently, we carried out a transcriptome-wide RNA-Seq gene expression analysis in ObPV- and PMMoV-inoculated pepper leaves. This analysis revealed that numerous pepper genes encoding transcription factors, including several WRKYs, especially the *CaWRKY2* gene, were strongly up-regulated by ObPV.

The present study was conducted with the aim of gaining a more detailed picture of the role of CaWRKY2 in plant–virus interactions. Firstly, we measured the effects of ObPV and PMMoV on the expression of *CaWRKY2* in infected pepper leaves. We also carried out a functional analysis of the *CaWRKY2* gene by its transient overexpression in *N. benthamiana*. In leaves transiently expressing the *CaWRKY2* gene, we measured the TMV multiplication rate and the expression of defense-related genes. In addition, we investigated the effect of *CaWRKY2* overexpression on TMV induced systemic necrosis.

## 2. Materials and Methods

### 2.1. Plants and Virus Inoculations

Seeds of the pepper (*Capsicum annuum* L.) cultivar TL 1791 harboring the *L^3^* resistance gene [29] were planted into soil and grown under greenhouse conditions (25 °C; photoperiod of 16 h; 160 μmol m^−2^ s^−1^ radiation; relative humidity of 75–80%). Two-month-old plants were used for the experiments. ObPV and PMMoV inoculations of pepper leaves were carried out as described earlier [22,23]. The ObPV strain was isolated in Hungary (formerly used synonym: Ob strain of tomato mosaic virus) (GenBank L11665 and NC_003852) [22]. The *L*^3^-resistance-breaking strain of PMMoV was isolated in Louisiana, USA (formerly used synonym: Samsun latent strain of tobacco mosaic virus) [22,30]. The genome of this PMMoV strain has not been sequenced yet. Mock inoculations were also carried out as controls (treatment without any virus to test the effect of the slight mechanical injury caused during virus inoculation). Following inoculations, the plants were kept in a growth chamber at 22 °C with a 16 h photoperiod. Total RNA content was extracted from pepper leaves at 4, 8, 24, 48, and 72 h post-inoculation (hpi) from the virus-inoculated leaves and from the corresponding mock-inoculated control leaves.

*N. benthamiana* plants were grown in a greenhouse for six weeks, and they were used to perform the transient overexpression of *CaWRKY2* by agroinfiltration. Four days after agroinfiltration, three middle leaves of *CaWRKY2*-overexpressing plants as well as of control plants carrying an empty expression vector were inoculated with the TMV-U_1_ strain. No abrasive was necessary for successful infection. In parallel experiments, *N. benthamiana* plants were also mock inoculated. TMV-infected and mock-inoculated control plants were kept in a growth chamber at 22 °C with 16/8 h light/dark cycles. Total RNA was extracted from *N. benthamiana* leaves after various time periods in all treatments.

### 2.2. RNA Extraction and Gene Expression Analysis by RT-PCR

Total RNA was extracted from infected and control pepper or *N. benthamiana* leaves. Leaf tissues (0.1 g) were ground under liquid nitrogen and RNA was extracted with an RNeasy Plant Mini Kit (Qiagen, Hilden, Germany). Reverse transcription (RT) of 2.5 μg total RNA was carried out with a RevertAid H Minus First Strand cDNA Synthesis kit (Thermo Fisher Scientific, Waltham, MA, USA) using an oligo(dT) primer. For the RT-PCR measurement of TMV coat protein gene transcript, RT was carried out with the reverse primer of the specific primer pair designed for the TMV coat protein gene (Table 1). PCRs were conducted with a PTC 200 DNA Engine extended with an ALS-1296 sample holder (Bio-Rad, Hercules, CA, USA) as described earlier [25]. The oligonucleotide primer pairs used in our studies are shown in Table 1. In the experiments with *N. benthamiana*, the expression of an actin gene served as a constitutive control (Table 1). Relatively low cycle numbers (22–28 cycles) were used to maintain initial differences in target transcript amounts (semiquantitative PCR conditions). Following PCR, 1% agarose gel was used to separate the reaction products, and GelRed nucleic acid stain (Biotium, Hayward, CA, USA) was used to visualize the products.

### 2.3. Analysis of Gene Expression Using Quantitative Real-Time RT-qPCR

To quantify the changes in *CaWRKY2* expression in virus-inoculated pepper leaves, RT-qPCR assays were conducted by using a DNA Engine Opticon 2 instrument (MJ Research, Waltham, MA, USA). The qPCR reaction mixture contained 2.5 μL of 10-fold diluted cDNA (0.75 μL of 5 μM), 0.375 μM of each primer (Table 1), and the iQ SYBR Green 2× Supermix (Bio -Rad, Hercules, CA, USA) in a final volume of 15 μL. The reaction parameters were as follows: initial denaturation at 95 °C for 6 min; and then 40 cycles of 95 °C for 30 s; 30 s at specific annealing temperatures, then 72 °C for 30 s; and finally, measurement of the fluorescence intensity of SYBRGreen dye at 84 °C for 15 s. After qPCR, the specificity of the product was checked by detecting a melting curve from 55 °C to 90 °C. The pepper ubiquitin-conjugating enzyme 3 gene (*CaUBI-3*) was selected as the housekeeping control gene (Table 1). Alterations in transcript abundance were calculated using the method of Livak and Schmittgen [33].

Changes in gene expression levels in *CaWRKY2*-overexpressing and control *N. benthamiana* leaves were analyzed by qPCR as described above. Specific primers are shown in Table 1. An actin gene served as a constitutive, housekeeping control gene (Table 1). All analyses were carried out in three independent experiments.

### 2.4. The Construction of Gateway Vectors Carrying the CaWRKY2 Gene

We constructed an entry vector and subsequently an expression vector carrying the CaWRKY2 gene (1647 bp) by using the Gateway vector system (Figure S1, Thermo Fisher Scientific, Waltham, MA, USA) [34,35,36]. As a first step, the open reading frame (ORF) of CaWRKY2 (GenBank: DQ402421), including the stop codon, was amplified by RT-PCR using an ORF-specific primer pair (5′ WRKY2-OSP: GGAGATAGAACC**ATGGCTGCTTCAAGTTTCTCAT**, 3′ WRKY2-OSP: CCTCCGGATCC**TCAGCAAAGCAATGACTCCATA**). These primers contain CaWRKY2-specific sequences (highlighted in bold letters) and linker sequences (normal letters) [37]. In this RT-PCR, total RNA extracted from ObPV-inoculated pepper leaves at 72 hpi was used. The PCR program consisted of using a temperature of 94 °C for 3 min, and then 35 cycles as follows: 94 °C for 1 min, annealing temperature (55 °C) for 1 min, 72 °C for 3 min, and finally, incubation at 72 °C for 10 min. The PCR product (the CaWRKY2 gene flanked by linker sequences) was separated on 0.5% agarose gel, purified by a Gene JET Gel Extraction Kit (Thermo Fisher Scientific, Waltham, MA, USA), and the resulting DNA sample was used as template for a second PCR.

The second PCR reaction was carried out with a universal primer pair (uni *att*B primers) containing linker sequences as well as specific adapter sequences (*att*B sites), which allow for the insertion of *CaWRKY2* into a Gateway vector (5′ uni *att*B1: GGGG**ACAAGTTTGTACAAAAAAGCAGGCT**TCGAAGGAGATAGAACCATG, 3′ uni *att*B2: GGGG**ACCACTTTGTACAAGAAAGCTGGGT**CACCGCCTCCGGATC). The *att*B sites are highlighted in bold letters, while the linker sequences are underlined. The PCR program consisted of using a temperature of 94 °C for 5 min, and then 35 cycles as follows: 94 °C for 1 min, annealing temperature (55 °C) for 1 min, 72 °C for 3 min, and finally, incubation at 72 °C for 10 min. The product of the second PCR reaction (the *CaWRKY2* gene flanked by two *att*B sites) was separated on a 0.5% agarose gel and purified. Next, the entry vector (pENTR) was constructed by transferring the product of the second PCR into the Gateway pDONR ZEO vector carrying two *att*P sites by using the Gateway BP clonase II enzyme mix (Thermo Fisher Scientific, Waltham, MA, USA). After recombination of the matching *att*B and *att*P sites, the *CaWRKY2* gene was flanked by two *att*L sites within the entry vector. The vector plasmids were introduced into competent *Escherichia coli* cells (TOP10 strain). The *E. coli* cells were spread on low-salt LB agar medium containing zeocin and incubated at 37 °C overnight, and then the positive colonies were selected by colony PCR with the plasmid-specific M13 primer pair (Table 1). The PCR program consisted of using a temperature of 94 °C for 2 min, and then 25 cycles as follows: 94 °C for 0:45 min, 52 °C for 0:45 min, 72 °C for 2:30 min, and finally, incubation at 72 °C for 10 min. From the positive *E. coli* TOP10 colonies, the entry vector plasmids carrying the *CaWRKY2* gene were purified by MiniPrep Express Matrix (MP Biomedicals, Irvine, CA, USA). The purified plasmid carrying *CaWRKY2* was sequenced to verify the cloning product.

From the purified entry vector, the *CaWRKY2* gene was shuttled into the pEarleyGate 100 vector carrying two *att*R sites (destination vector, pDEST) by using the Gateway LR clonase II enzyme mix (Thermo Fisher Scientific, Waltham, MA, USA). This vector contains CaMV 35S constitutive promoter (cauliflower mosaic virus 35S RNA promoter) and OCS terminator (Octopine synthase terminator). After recombination of the matching *att*L and *att*R sites, *CaWRKY2* was again flanked by *att*B sites in the resulting expression vector [34,36]. The success of cloning was verified by PCR, and the expression vector plasmid was introduced into competent *E. coli* cells using heat shock and then propagated and purified as described above. For ‘empty vector’ control plants, the empty pEarlyGate 100 vector was used without *CaWRKY2*.

### 2.5. Agroinfiltration of N. benthamiana Leaves

Purified plasmids carrying the expression vector containing the *CaWRKY2* gene as well as the empty expression vector as control were transferred into *A. tumefaciens* by electroporation. The *A. tumefaciens* LBA4404 strains carrying the plasmid pEarleyGate 100 containing *CaWRKY2* or the empty expression vector were grown overnight on LB plates at 27 °C supplemented with appropriate antibiotics. The bacteria were suspended in infiltration buffer (1.95 g MES (2-[N-morpholino]ethanesulfonicacid) and 2 g MgCl_2_.6 H_2_O in 1 L distilled water, pH 5.6). Bacterial cell densities were adjusted with a spectrophotometer to an optical density of 0.4 at 600 nm (OD_600_) and supplemented with acetosyringone (final concentration 150 µM). After three hours of incubation at room temperature, the bacterial suspensions were injected into plant leaves using a needleless syringe.

Three middle leaves of two-month-old *N. benthamiana* plants were infiltrated with *A. tumefaciens* carrying the plasmid containing *CaWRKY2* as well as with *A. tumefaciens* carrying the empty expression vector. The transformed plants were kept in growth chambers at 22 °C with 16 h illumination/8 h dark conditions. For gene expression studies, total RNA was extracted from the transformed leaves at various time periods after transformation.

### 2.6. Statistical Analysis

Generally, three independent biological experiments were carried out. Numerical data represent the mean of three independent parallel experiments ± standard error. The significant difference between mean values obtained in virus-inoculated and mock-inoculated control leaves were evaluated by Student’s *t*-test. Differences were considered to be significant at *p* < 5%.

## 3. Results

### 3.1. The Effects of ObPV and PMMoV on the Expression of CaWRKY2 in Infected Pepper Leaves

**Figure 1:**
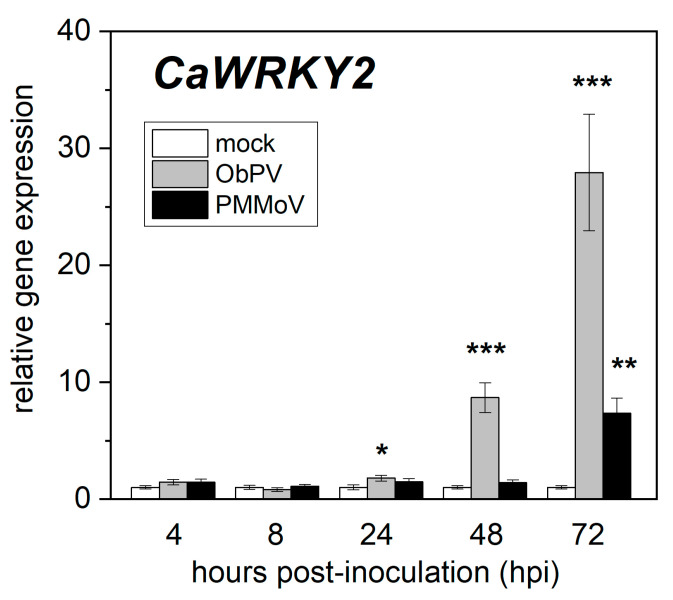
The effects of Obuda pepper virus (ObPV), pepper mild mottle virus (PMMoV), and mock-inoculations on the transcript abundance of the *CaWRKY2* gene in inoculated pepper leaves at various time points following inoculation as detected by RT-qPCR with the specific primer pair *CaWRKY2* (Table 1). The expression of a ubiquitin gene (*UBI-3*) was examined as a control housekeeping gene. The PCR conditions and GenBank accession numbers of all genes are shown in Table 1. The open, gray, and black columns represent mock-, ObPV-, and PMMoV-inoculated leaves, respectively. The mean values of three independent experiments ± SD are shown. The symbols *, **, and *** show significant differences between mock- and virus-inoculated plants at *p* < 5%, < 1%, and < 0.1%, respectively.

The ObPV inoculation of pepper leaves resulted in the appearance of necrotic lesions (hypersensitive reaction) at 3 days post-inoculation, while PMMoV-inoculated leaves showed no visible symptoms, in accordance with earlier studies [22,23]. Total RNA was extracted from ObPV-, PMMoV-, and mock-inoculated pepper leaves at 4, 8, 24, 48, and 72 hpi, and changes in the expression of *CaWRKY2* were analyzed by RT-qPCR. ObPV inoculation led to a significant induction of *CaWRKY2* expression at 24 hpi, and the expression gradually rose further, reaching a 28-fold increase at 72 hpi as compared to the mock control (Figure 1). In contrast, PMMoV caused only a late and weak increase in *CaWRKY2* expression (7-fold induction at 72 hpi), while mock inoculation did not show any effect (Figure 1). These results are in accordance with our earlier RNA-Seq results [5].

### 3.2. Overexpression of CaWRKY2 in Transformed N. benthamiana Leaves

We constructed a Gateway expression vector containing the entire coding sequence of *CaWRKY2*, including its stop codon. The PCR products were cloned into the pEarleyGate 100 vector driven by the constitutive CaMV 35S promoter. The *A. tumefaciens* strain carrying the *CaWRKY2* gene was infiltrated into the leaves of mature *N. benthamiana* plants. To verify the success of this genetic transformation, total RNA was isolated from *N. benthamiana* leaves transformed with the vector carrying *CaWRKY2* or the empty expression vector at various time points after transformation.

**Figure 2:**
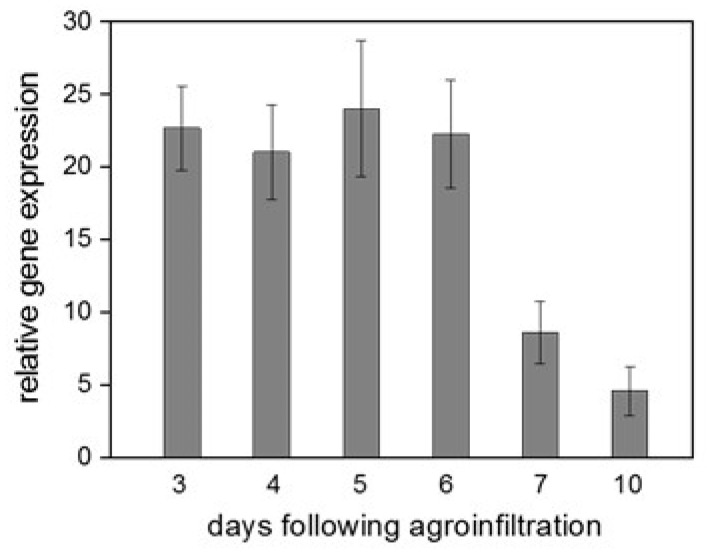
Expression of *CaWRKY2* gene in transiently transformed *N. benthamiana* leaves. *CaWRKY2* expression detected by RT-qPCR 3–10 days after agroinfiltration with specific primer pair CaWRKY2rt (Table 1). Means of three independent parallel experiments ± SD are shown.

By using the total RNA extracts, RT-qPCR analyses were carried out to detect the expression of *CaWRKY2*. The RT-qPRC experiments showed that *CaWRKY2* was strongly expressed 3 days after agroinfiltration; the expression remained high from 3 to 6 days, and then it sharply fell at 7 and 10 days (Figure 2). No *CaWRKY2* expression was observed in the *N. benthamiana* leaves transformed with the empty expression vector. These results indicate that transient gene expression was successful even though we did not use any silencing suppressor because we wanted to infect the transformed leaves by TMV in later experiments.

### 3.3. TMV Inoculation of N. benthamiana Leaves Expressing CaWRKY2

Leaves of *N. benthamiana* plants transformed with an expression vector carrying *CaWRKY2* or with an empty expression vector were infected with the TMV-U_1_ strain 4 days after agroinfiltration. In parallel control experiments, mock inoculations were also performed. Upon TMV inoculation, diffuse necrotic areas appeared on the inoculated leaves 3 days post-inoculation (dpi), in accordance with earlier observations [38]. Later, the virus spread into the vascular tissues, resulting in the bending of the plant stem. Ultimately, systemic necrosis developed, which led to plant death at 7 dpi, as described earlier [38]. The transient overexpression of *CaWRKY2* did not significantly affect the number of necrotic lesions on the TMV-inoculated leaves (results not shown). There were no visible lesions on the mock-inoculated leaves.

**Figure 3:**
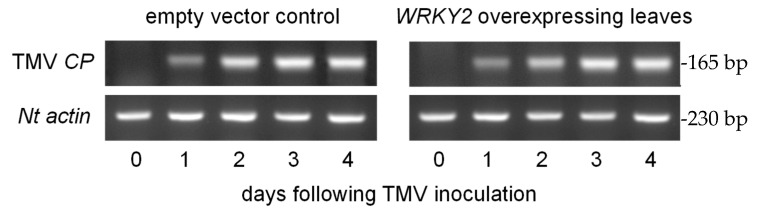
The monitoring of TMV replication in *N. benthamiana* leaves overexpressing *CaWRKY2* and in control leaves carrying an empty expression vector at various time points after TMV inoculation. The expression of the coat protein gene (*CP*) of TMV was detected by RT-PCR with the specific primer pair TMV-CP (Table 1). The expression of an actin was also detected as a constitutive control gene. The representative results of three independent parallel experiments are shown.

In the next experiments, we wanted to examine whether the multiplication rate of TMV in the inoculated *N. benthamiana* leaves is modified by the transient overexpression of the *CaWRKY2* gene. Four days after agroinfiltration, the leaves expressing *CaWRKY2* or carrying the empty vector were inoculated with TMV, and the replication rates of TMV were compared between the two treatments at various time points after TMV inoculation. TMV replication was monitored by measuring the transcript abundance of the TMV coat protein gene (*CP*) by semi-quantitative RT-PCR using the specific primer pair *TMV-CP* (Table 1). The transcript abundance of *TMV-CP* markedly increased from 1 dpi to 4 dpi in both the *CaWRKY2*-overexpressing and empty control leaves (Figure 3). However, the overexpression of *CaWRKY2* did not alter the multiplication of TMV in the inoculated *N. benthamiana* leaves (Figure 3). The expression of the constitutive control actin gene was not modified by TMV either (Figure 3).

### 3.4. Induction of Defense Genes in N. benthamiana Leaves by TMV Inoculation

**Figure 4:**
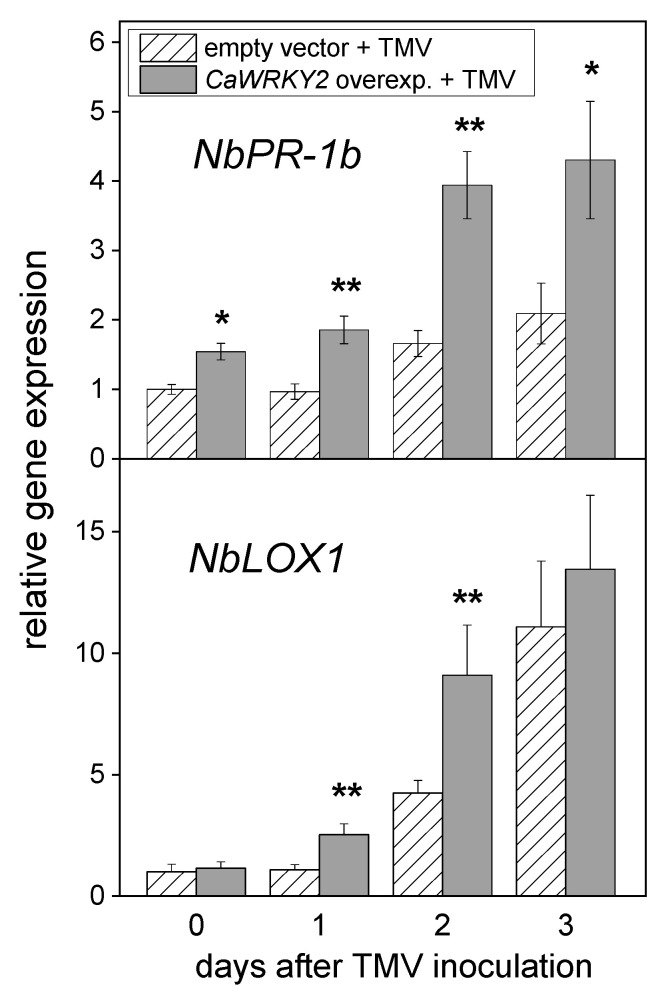
The effect of TMV inoculation on the transcript abundance of *PR-1b* and *LOX1* genes in *N. benthamiana* leaves overexpressing *CaWRKY2* and in empty vector control leaves at various time points after TMV inoculation, as detected by RT-qPCR. The expression values were normalized to those of the constitutive control actin gene (Table 1) and then related to empty vector leaf samples at 0 dpi. The mean values of three independent experiments ± SD are shown. The symbols * and ** show significant differences between *CaWRKY2*-overexpressing and empty vector control plants at *p* < 5% and <1%, respectively.

We wanted to explore the effects of TMV inoculation on the expression of defense genes in *N. benthamiana* leaves overexpressing *CaWRKY2* as well as in control leaves carrying an empty expression vector. Nine defense-related *N. benthamiana* genes were used for the experiments: two 1-aminocyclopropane-1-carboxylate synthases (*ACS2* and *ACS6*), two glutathione S-transferases (*GSTF* and *GSTU1*) [31], a 9-lipoxygenase (*LOX1*), three pathogenesis-related genes (*PR-1a*, *PR-1b*, and *PR10*), and a WRKY gene (*WRKY1*). The expression of these genes was examined by semi-quantitative RT-PCR with specific and degenerate primer pairs (Table 1). TMV inoculation markedly induced the expression of two genes (*LOX1* and *PR-1b*) but did not significantly influence the expression of *ACS2*, *ACS6*, *GSTF*, *GSTU1*, *PR-1a*, *PR10*, and *WRKY1*. The expression of a tobacco actin, examined as a household control gene, was not significantly altered by either virus inoculation or by mock inoculation. The expression levels of *LOX1* and *PR-1b* were also examined by RT-qPCR with specific primer pairs (Table 1). TMV inoculation significantly increased the expression of both genes in *N. benthamiana* leaves overexpressing *CaWRKY2* as compared to TMV-inoculated control leaves carrying an empty expression vector (Figure 4). At 2 days post TMV inoculation, the expression of the *PR-1b* and *LOX1* genes were 2.4-fold and 2.1-fold higher in the *CaWRKY2* overexpressing leaves than in the empty vector control leaves, respectively (Figure 4). Interestingly, the expression of *PR-1b* was stronger in *N. benthamiana* leaves overexpressing *CaWRKY2* than in control leaves carrying an empty expression vector, also without TMV inoculation (at 0 dpi) (Figure 4).

### 3.5. Effect of Overexpression of CaWRKY2 on TMV-Induced Systemic Necrosis

**Figure 5:**
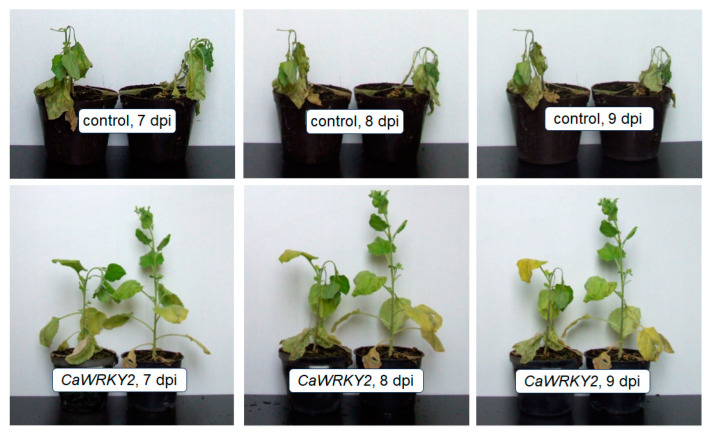
Systemic necrosis caused by TMV inoculation in *N. benthamiana* plants transformed with *CaWRKY2* gene or with empty expression vector at 7, 8, and 9 days after TMV inoculation (dpi). TMV inoculation was performed on three previously agroinfiltrated leaves. Experiment was performed in six replicates with same results.

TMV infection is known to result in systemic necrosis, complete plant death, in *N. benthamiana* approx. 7 days after TMV inoculation [38]. We investigated the effect of *CaWRKY2* overexpression on this rapid systemic necrosis by visual observation in six independent experiments. Although the transient expression of *CaWRKY2* did not modify the local effects of TMV inoculation in the inoculated leaves, *CaWRKY2* overexpression had an impact on the systemic effects of TMV. We observed a rapid systemic necrosis in the case of empty vector control *N. benthamiana* plants, causing complete death of the plant one week after TMV infection as expected (Figure 5). However, intriguingly, the transient overexpression of *CaWRKY2* delayed the systemic necrosis by about 3 days, and plant death occurred approximately 10 days post TMV inoculation (Figure 5). Nevertheless, the transient overexpression of *CaWRKY2* was not able to prevent TMV-inoculated systemic death at later time points. No local or systemic symptoms were observed in the mock-inoculated plants.

## 4. Discussion

Transcriptional reprogramming in virus-infected plants is an essential element of their resistance to viruses. This process is largely mediated by transcription factor proteins. Transcription factors can up- or down-regulate a large number of target genes simultaneously [39]. The identification of those transcription factors, which are rapidly and strongly activated, specifically in resistant plants, may provide novel insights into the molecular mechanisms of antiviral resistance. For our investigations, we selected the pepper WRKY2 transcription factor that belongs to group I of WRKYs (GenBank accession DQ402421; [20]). Interestingly, in the N-terminal region of the CaWRKY2 protein sequence, five Ser-Pro motifs can be found, which form a cluster (SP-cluster). Within the SP cluster, Ser residues can serve as phosphorylation sites for MAP kinase (MAPK) enzymes. Adjacent to the SP-cluster, a MAPK docking site (D-domain) was also identified in CaWRKY2, which presumably facilitates the interaction between the MAPK and WRKY proteins [40]. Phosphorylation of the SP cluster in the NbWRKY8 protein increased both its DNA binding and its transcriptional activity [41]. An SP cluster and a D-domain were also identified in the pepper WRKY-a sequence [8]. MAPK cascades can activate WRKYs not only post-transcriptionally but also transcriptionally [40,42].

In our initial experiments, we compared the changes in the expression of the *CaWRKY2* gene between resistant and susceptible pepper–tobamovirus interactions by RT-qPCR. ObPV inoculation of a pepper cultivar harboring the *L*^3^ resistance gene (incompatible interaction) led to the rapid and robust up-regulation of *CaWRKY2*. In contrast, PMMoV inoculation of the same pepper cultivar (compatible interaction) resulted only in a weak and slow induction of *CaWRKY2* (Figure 1). These expression patterns suggest that *CaWRKY2* may play an important role in antiviral resistance. However, the exact function of *CaWRKY2*, like that of most WRKYs, is still elusive. Therefore, we decided to use a quick and simple method for the functional analysis of *CaWRKY2,* transiently overexpressing this gene in *N. benthamiana* leaves. Transient overexpression mediated by *A. tumefaciens* infiltration (agroinfiltration) is often employed as an alternative to stable transformants due to its rapidity and efficiency [43,44,45]. In our experiments, the transient overexpression of *CaWRKY2* in *N. benthamiana* leaves proved to be straightforward and successful (Figure 2).

To learn more about the role of CaWRKY2 in antiviral plant defense, we inoculated *N. benthamiana* leaves overexpressing *CaWRKY2* as well as control leaves with TMV. The expression of *CaWRKY2* did not influence the local symptoms of TMV nor the multiplication rate of the virus in the inoculated leaves (Figure 3).

In the following experiments, we investigated the effect of TMV inoculation on the expression of various defense genes in *N. benthamiana* leaves overexpressing *CaWRKY2*. The expression of *LOX1* and *PR-1b* was up-regulated by TMV inoculation in both the *CaWRKY2*-overexpressing leaves and in the control, empty vector leaves (Figure 4). However, the TMV-induced up-regulation of both genes was stronger in the *CaWRKY2*-overexpressing leaves than in the empty vector leaves (Figure 4). Interestingly, the overexpression of *CaWRKY2* led to a significantly elevated expression of *PR-1b* also without TMV inoculation (Figure 4). Prior to our studies, it was already known that silencing *WRKY* genes resulted in the suppression of *LOX* expression in various plants, which showed that these WRKYs positively regulated *LOX* expression [8,46]. Pathogenesis-related 1 (PR-1) proteins, including PR-1b, were identified as TMV-inducible proteins in tobacco many years ago [47]. Despite long years of research work, their exact function is still elusive [9,48]. The constitutive overexpression of the *PR-1b* gene in tobacco leaves did not modify their resistance against TMV [49]. Nevertheless, the expression of *PR-1* genes is often investigated as a marker of systemic acquired resistance (SAR) [9].

It is well known that TMV inoculation of *N. benthamiana* leaves leads to lethal systemic necrosis and plant death 7 days post-inoculation [38,50]. Recent studies showed that phloem is a key responsive tissue during TMV infection [51]. In our experiments, the transient overexpression of *CaWRKY2* in *N. benthamiana* leaves delayed (by approx. 3 days) the systemic necrosis caused by TMV (Figure 5). However, it was ultimately observed that *CaWRKY2* was unable to prevent plant death. It is conceivable that the overexpression of *WRKY2* inhibits the cell-to-cell movement of TMV that occurs through the plasmodesmata. Overexpression of the *NbWRKY40* gene (II-a group WRKY) in *N. benthamiana* led to an increased deposition of callose in the neck of plasmodesmata via the elevation of endogenous salicylic acid levels. Presumably, this effect contributed to the elevated resistance of these mutant plants against tomato mosaic virus (ToMV) [9]. We hypothesize that in our experiments, CaWRKY2 activates biochemical reactions that hinder the movement of TMV into the vascular tissues or into systemic leaves. In our future experiments, we will therefore investigate the effects of transformation with *CaWRKY2* in the vascular tissues or in the systemic leaves of TMV-infected *N. benthamiana* plants.

## Figures and Tables

**Table 1:** Sequences of primer pairs in 5′ to 3′ directions used for semi-quantitative PCR and qPCR investigations. Gene identification numbers are shown in parenthesis (databases: NCBI and Solanaceae Genomics Network).

Target Gene	Forward Primer	Reverse Primer	Product Length (bp)	Annealing Temperature (°C)
*CaWRKY2* (DQ402421)	accactgttacggagggtgt	cgaacgaaaggaaactgcta	182	58
*CaWRKY2rt* (DQ402421)	gcacaagtccaggatgtcca	tgagatgtcacggagggtct	205	60
M13	gtaaaacgacggccag	caggaaacagctatgac	n.d.	52
*TMV-CP* (AF165190)	cttgtcatcagcgtgggc	aagtcactgtcagggaac	165	47
*NbACS2* (Niben101Scf09512g03008)	aggtttgtgggtgttaagaa	ctaattcctccagtttaagtt	191	58
*NbACS6* (Niben101Scf02334g00004)	aggagcaaacttcagatcag	ctgcacaaaatgggataa	231	58
*NbGSTF* (degenerate primers) *	ctggkgawcacaagaagc	gccaaratatcagcacacc	n.d.	50
*NbGSTU1* (degenerate primers) *	gatggcagaagtgaagttg	ctcctagccaaaatscca	n.d.	50
*NbLOX1* (KC585517)	gcctgttaaagttccatata	gcctacagcattacatcc	231	58
*NtPR-1a* (D90196)	taaaaagcaacttaaagtcaa	caagtagctagaccatcaaca	194	58
*NtPR-1b* (X03465)	cagggaagtggcgattttatg	agaccacttggactttttacagat	400	60
*NbPR-10* (KF841443)	cagtgaaggcaaagatcaagc	caagcccttaggaactcttag	253	60
*NbWRKY1* (Niben101Scf02430Ctg025)	ctcgtcggggtcttacatga	ttacagctgccaaccaatct	277	60
*CaUBI-3* (AY486137	tgtccatctgctctctgttg	caccccaagcacaataagac	204	60
*Nt actin* (X69885) **	cggaatccacgagactacatac	gggaagccaagatagagc	230	60

* [31]; ** [32], 1993; n.d. = not defined.

## Data Availability

All of the available data are presented in the manuscript.

## Supplementary Materials

The following supporting information can be downloaded at https://www.mdpi.com/article/10.3390/life15040669/s1. Figure S1. Construction of Gateway vectors carrying CaWRKY2 gene.
