# Supplementary material for: Transient Overexpression of the Pepper WRKY2 Gene in Nicotiana benthamiana Markedly Delays the Systemic Necrosis Caused by Tobacco Mosaic Virus
Source: Life (Basel). 2025 Apr 17;15(4):669. doi: 10.3390/life15040669 (PMC12028993; doi:10.3390/life15040669)

Figure S1. Construction of Gateway vectors carrying CaWRKY2 gene

1. RT-PCR with CaWRKY2 specific primers (WRKY2-OSP) containing part of the *att* B sequences.

2. PCR with uni *att* B primers to obtain complete *att* B.

3. BP clonase reaction with pDONR ZEO vector, resulting in pENTR vector.

4. LR clonase reaction with pEarleyGate 100 vector resulting in expression vector.

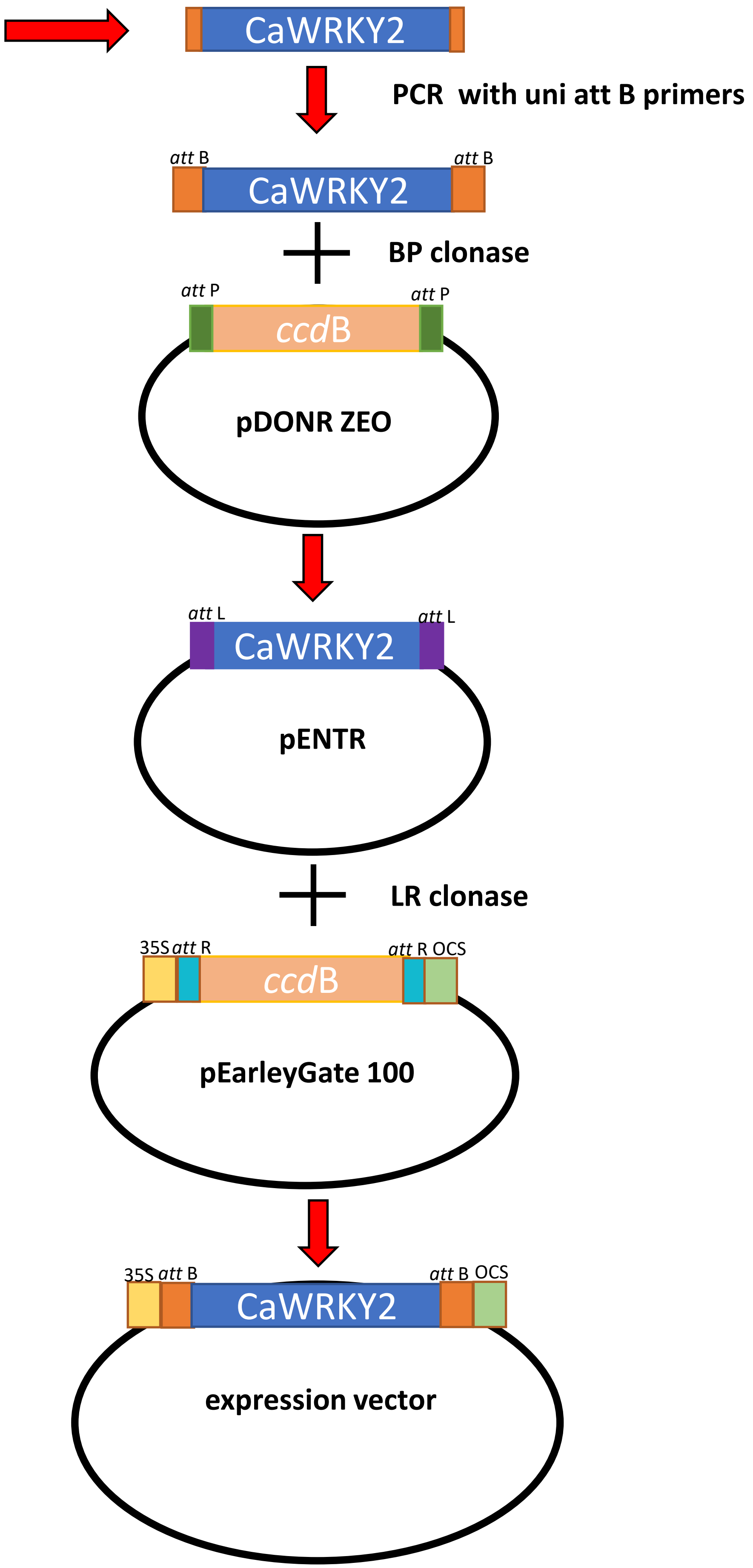

Supplement: Supplementary file 1 [file life-15-00669-s001.zip › life-3496300-supplementary.pdf]
